# Supplementary material for: Inferring protein fitness landscapes from laboratory evolution experiments
Source: PLoS Comput Biol. 2023 Mar 1;19(3):e1010956. doi: 10.1371/journal.pcbi.1010956 (PMC10010530; doi:10.1371/journal.pcbi.1010956)
Supplement: S1 Fig — (PDF) [file pcbi.1010956.s001.pdf]

Supplementary Information:
Inferring protein fitness landscapes from laboratory evolution experiments

Supplementary Figures

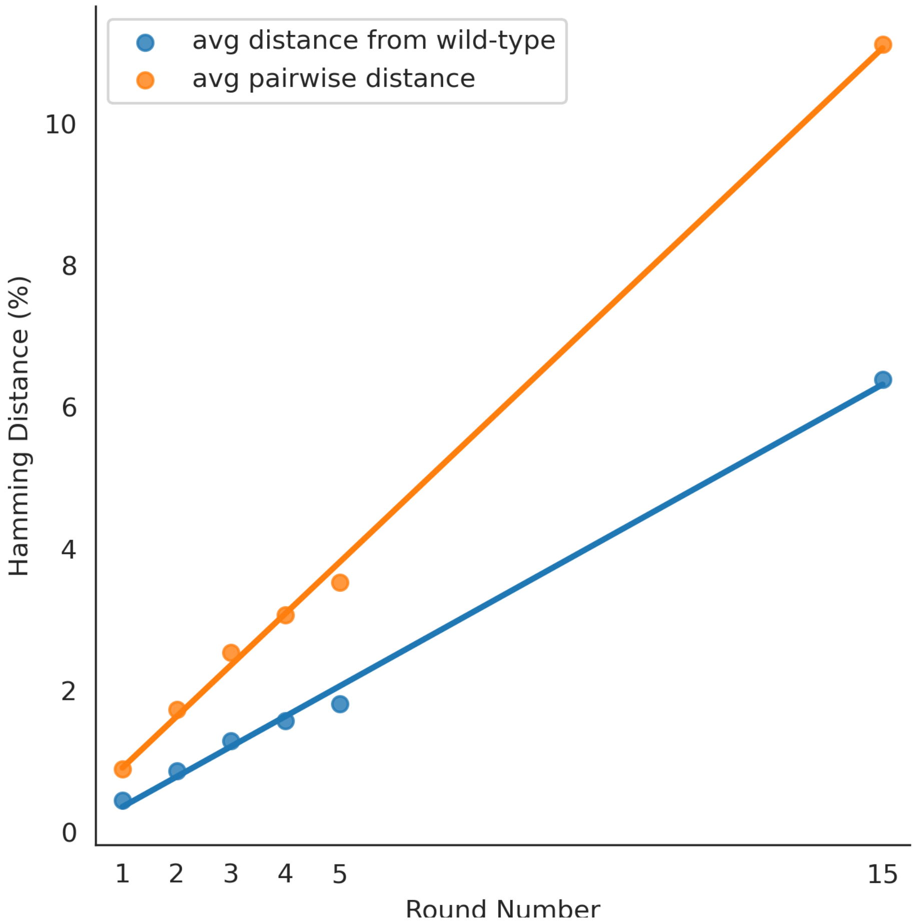

Figure S1. Average Hamming distance from wild-type and average pairwise Hamming distance between sequences per round showing a roughly linear progression
