# Supplementary material for: Inferring protein fitness landscapes from laboratory evolution experiments
Source: PLoS Comput Biol. 2023 Mar 1;19(3):e1010956. doi: 10.1371/journal.pcbi.1010956 (PMC10010530; doi:10.1371/journal.pcbi.1010956)
Supplement: S2 Fig — (PDF) [file pcbi.1010956.s002.pdf]

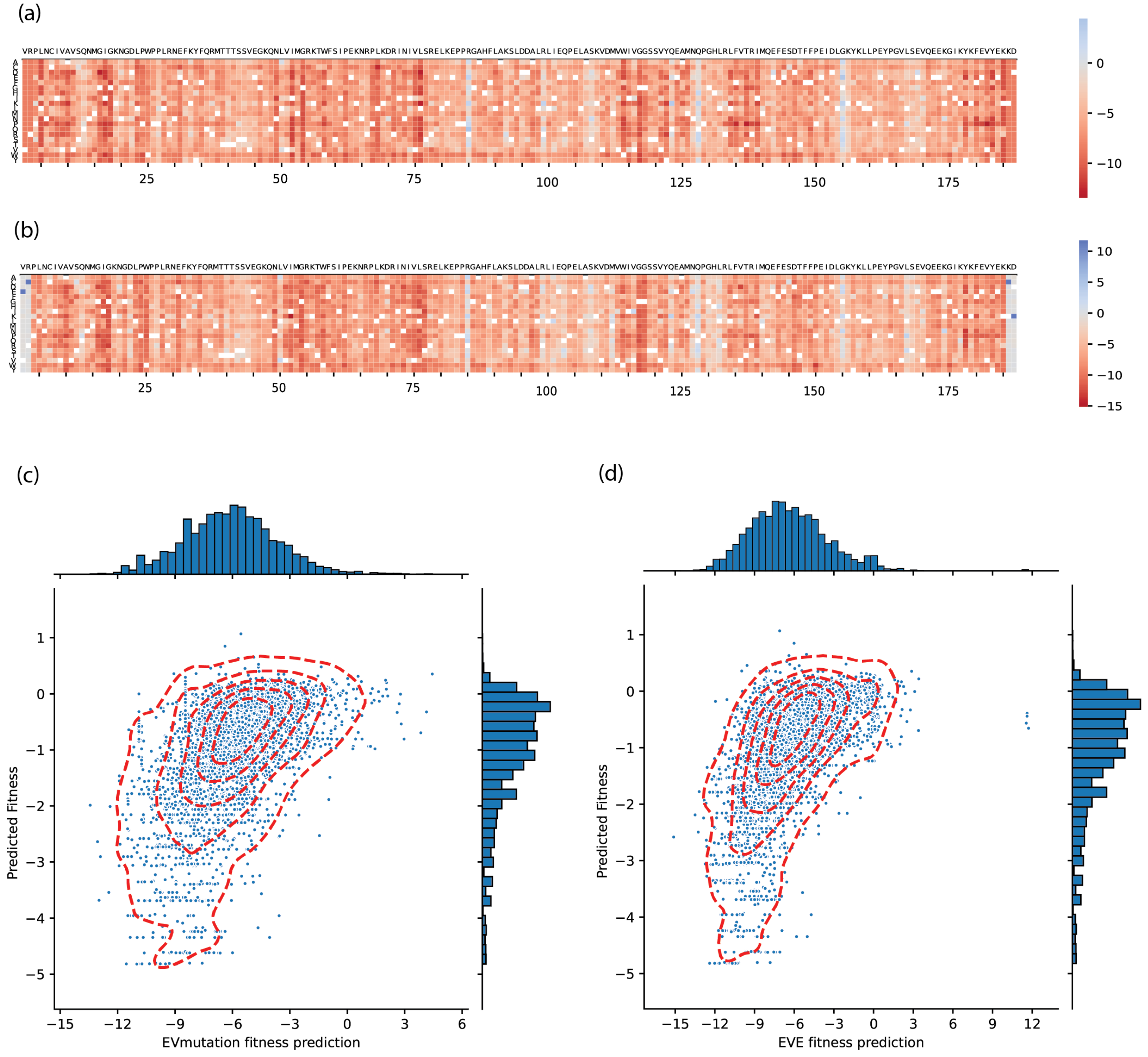

Figure S2. Mutation effect prediction on natural DHFR sequences implemented using the (a) EVmutation [1] (Potts DCA) and (b) EVE [2] (Bayesian VAE) programs. Mutation effect prediction from Main Fig 2a plotted against (c) EVmutation mutation effect prediction (Spearman correlation 0.54) and (d) EVE (Spearman correlation 0.62). Red dotted lines show contours of kernel density estimates of the joint density.
