# Supplementary material for: Inferring protein fitness landscapes from laboratory evolution experiments
Source: PLoS Comput Biol. 2023 Mar 1;19(3):e1010956. doi: 10.1371/journal.pcbi.1010956 (PMC10010530; doi:10.1371/journal.pcbi.1010956)
Supplement: S3 Fig — (PDF) [file pcbi.1010956.s003.pdf]

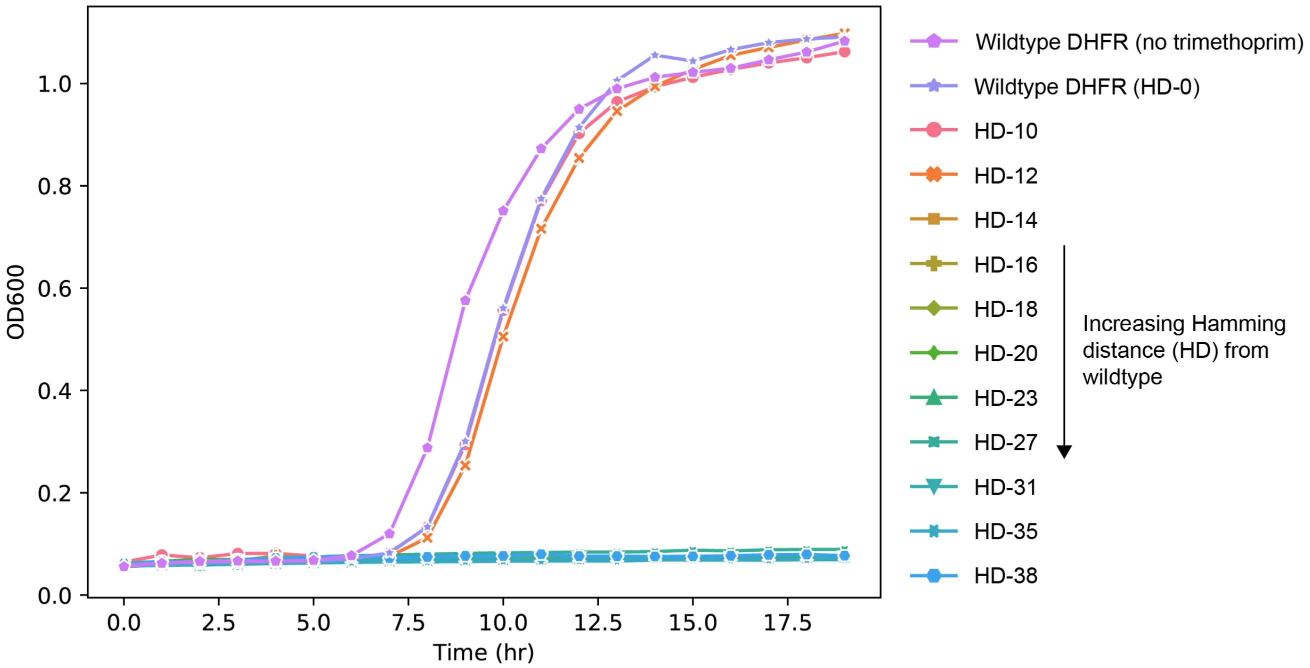

Figure S3. Growth curves of sequences designed using the inferred model, the most common sequence in round 15 and the wild-type sequence mDHFR.
