# Supplementary material for: Inferring protein fitness landscapes from laboratory evolution experiments
Source: PLoS Comput Biol. 2023 Mar 1;19(3):e1010956. doi: 10.1371/journal.pcbi.1010956 (PMC10010530; doi:10.1371/journal.pcbi.1010956)
Supplement: S4 Fig — (PDF) [file pcbi.1010956.s004.pdf]

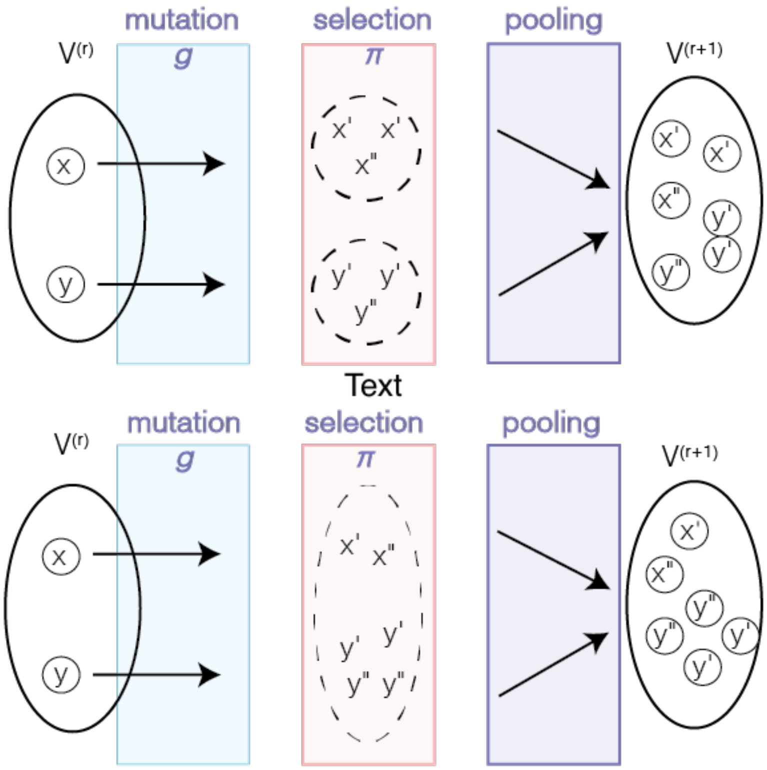

Figure S4. Diagram of an Idealized Experiment (first row) vs Real Experiment (second row). Suppose an overall capacity in the selection step is 6, and the relative fitness level of sequences x', x'', y', y'' is $x'' < x' \ll y'' < y'$. We see more x', x'' are present in $V^{(r+1)}$ in the first experiment due to a localized competition in (x', x'') and (y', y'').
