# Supplementary material for: Inferring protein fitness landscapes from laboratory evolution experiments
Source: PLoS Comput Biol. 2023 Mar 1;19(3):e1010956. doi: 10.1371/journal.pcbi.1010956 (PMC10010530; doi:10.1371/journal.pcbi.1010956)
Supplement: S5 Fig — (PDF) [file pcbi.1010956.s005.pdf]

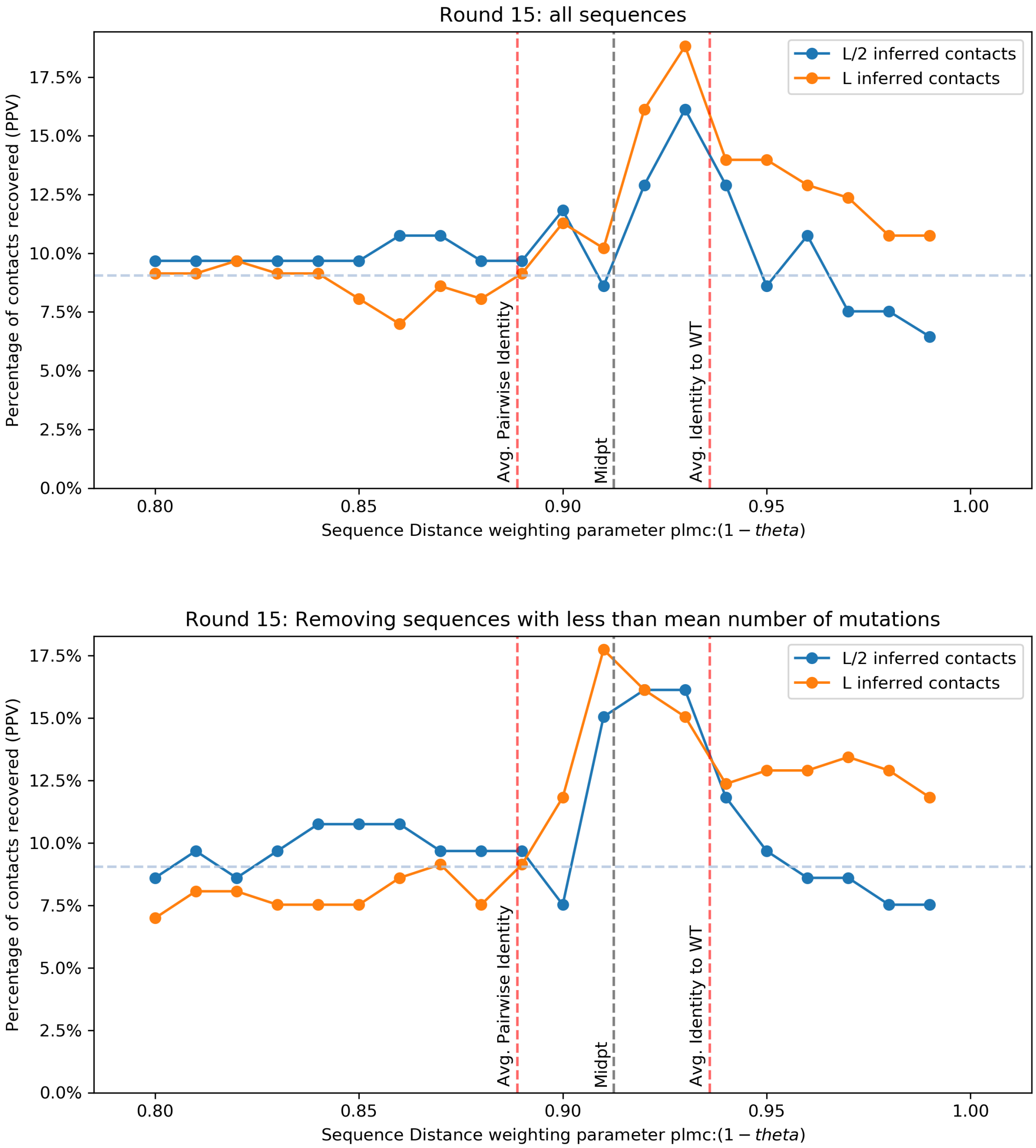

Figure S5. DCA methods trained on round 15 DHFR evolution data with different sequence weighting parameters $(1-\text{theta})$. We ran *plmc* [1] on all sequences (top panel). Also, we follow the reweighting method of [3] (bottom panel). We first remove sequences at a distance less than the average distance from wild-type. Then we find that setting the sequence weighting parameter to the midpoint of the pairwise identity and the average identity to wild-type results is optimal in that it results in the highest positive predicted value when looking at the top L contact predictions and close to optimal when looking at the top $L/2$ predictions.
