# Supplementary material for: Inferring protein fitness landscapes from laboratory evolution experiments
Source: PLoS Comput Biol. 2023 Mar 1;19(3):e1010956. doi: 10.1371/journal.pcbi.1010956 (PMC10010530; doi:10.1371/journal.pcbi.1010956)
Supplement: S1 Table — (PDF) [file pcbi.1010956.s006.pdf]

Supplementary Tables

Table S1. Estimate of Colony Formining Units and Fractional Functional

Iteration	CFUs	Fraction Functional (FF)	Functional Variants (FV)
1	1.61e+06	0.841	1.35e+06
2	6.84e+05	0.364	2.49e+05
3	1.02e+06	0.227	2.32e+05
4	1.07e+06	0.364	3.89e+05
5	5.74e+05	0.182	1.04e+05
6	1.55e+06	0.409	6.34e+05
7	4.50e+05	0.250	1.13e+05
8	6.00e+05	0.250	1.50e+05
9	2.32e+05	0.205	4.76e+04
10	4.04e+05	0.231	9.32e+04
11	3.94e+05	0.314	1.24e+05
12	2.48e+05	0.316	7.83e+04
13	1.44e+06	0.391	5.63e+05
14	1.06e+06	0.214	2.27e+05
15	7.08e+05	0.393	2.78e+05
