# Supplementary material for: Inferring protein fitness landscapes from laboratory evolution experiments
Source: PLoS Comput Biol. 2023 Mar 1;19(3):e1010956. doi: 10.1371/journal.pcbi.1010956 (PMC10010530; doi:10.1371/journal.pcbi.1010956)
Supplement: S2 Table — (PDF) [file pcbi.1010956.s007.pdf]

Table S2. The top 20 long range interaction scores between pairs of residues. The columns AA1 and AA2 refer to the wild-type amino acid at the corresponding residue. We label the distance between the residues in the 3D structure. We also label whether the residue is involved in the function of the protein by labeling nucleotide binding regions or an isolated nucleotide binding site of the protein. Many of the top 20 interactions involve functional residues.

	Res1	Res2	AA1	AA2	Residue distance	Frobenius Score	Contact	Res1 Function	Res2 Function
1	71	117	R	G	46	0.000208	no contact	Binding Site	Binding Region
2	25	71	W	R	46	0.000175	no contact	None	Binding Site
3	71	114	R	W	43	0.000164	5-8Å	Binding Site	None
4	25	117	W	G	92	0.000162	no contact	None	Binding Region
5	58	71	W	R	13	0.000157	5-8Å	None	Binding Site
6	25	114	W	W	89	0.000138	no contact	None	None
7	25	58	W	W	33	0.000131	no contact	None	None
8	58	117	W	G	59	0.000127	5-8Å	None	Binding Region
9	58	114	W	W	56	0.000123	no contact	None	None
10	67	117	P	G	50	0.000120	no contact	None	Binding Region
11	25	67	W	P	42	0.000118	no contact	None	None
12	67	114	P	W	47	0.000110	no contact	None	None
13	25	39	W	T	14	0.000109	no contact	None	None
14	25	118	W	G	93	0.000102	no contact	None	Binding Region
15	25	137	W	T	112	0.000102	5-8Å	None	None
16	39	114	T	W	75	0.000102	<5Å	None	None
17	58	67	W	P	9	0.000102	<5Å	None	None
18	25	68	W	L	43	0.000099	no contact	None	None
19	25	54	W	G	29	0.000098	no contact	None	None
20	39	71	T	R	32	0.000095	<5Å	None	Binding Site
