# Supplementary material for: Inferring protein fitness landscapes from laboratory evolution experiments
Source: PLoS Comput Biol. 2023 Mar 1;19(3):e1010956. doi: 10.1371/journal.pcbi.1010956 (PMC10010530; doi:10.1371/journal.pcbi.1010956)
Supplement: S3 Table — (PDF) [file pcbi.1010956.s008.pdf]

Table S3. Positive predictive value (PPV) of long range contacts recovered by different methods. These results are a table version of Figure 2f). $L = 186$

Number of predictions	Method	<5Å	5-8Å	<8Å
L/5	Round 15 reweight	2.70%	2.70%	5.41%
	Round 15 equal weight	2.70%	5.41%	8.11%
	Entire trajectory (this paper)	13.51%	16.22%	29.73%
L/2	Round 15 reweight	2.15%	13.98%	16.13%
	Round 15 equal weight	1.08%	5.38%	6.45%
	Entire trajectory (this paper)	10.75%	11.83%	22.58%
L	Round 15 reweight	5.38%	11.83%	17.20%
	Round 15 equal weight	2.15%	7.53%	9.68%
	Entire trajectory (this paper)	10.75%	12.90%	23.66%
2L	Round 15 reweight	4.57%	8.60%	13.17%
	Round 15 equal weight	3.49%	8.06%	11.56%
	Entire trajectory (this paper)	7.53%	13.17%	20.70%
