# Supplementary material for: Inferring protein fitness landscapes from laboratory evolution experiments
Source: PLoS Comput Biol. 2023 Mar 1;19(3):e1010956. doi: 10.1371/journal.pcbi.1010956 (PMC10010530; doi:10.1371/journal.pcbi.1010956)
Supplement: S4 Table — (PDF) [file pcbi.1010956.s009.pdf]

Table S4. Error-prone PCR mutation bias. The transitions are given from columns to rows i.e. the probability of mutation from nucleotide A to C is given in column A and row C.

transition	A	C	G	T
A	0.98860	0.00068	0.00205	0.00615
C	0.00110	0.99707	0.00021	0.00415
G	0.00415	0.00021	0.99707	0.00110
T	0.00615	0.00205	0.00068	0.98860
