# Supplementary material for: Inferring protein fitness landscapes from laboratory evolution experiments
Source: PLoS Comput Biol. 2023 Mar 1;19(3):e1010956. doi: 10.1371/journal.pcbi.1010956 (PMC10010530; doi:10.1371/journal.pcbi.1010956)
Supplement: S1 Text — (PDF) [file pcbi.1010956.s010.pdf]

Supplementary Methods and Mathematical Details

Markov chain approximation to infinite population dynamics

For each sequence in a fixed landscape Ω, we model the dynamics of the prevalence (or concentration) in each round as a mutation selection process and can be recursively written [4, 5] as

$$p^{(r+1)}(x) = \frac{\sum_{u\in\Omega} p^{(r)}(u) g(u \to x)\pi(x)}{\sum_{v,z\in\Omega} p^{(r)}(v) g(v \to z)\pi(z)} \tag{1}$$

The product in the numerator represents the number of sequences of x produced by sequence u as it is the product of the prevalence $p^{(r)}(u)$ of sequence u in round r, the mutation probability $g(u \to x)$ from sequence u to x and the fitness level $\pi(x)$ of sequence x. The denominator represents the total production of new sequences produced in round $r+1$ and it scales the prevalence vector $p^{(r+1)}$ so that it sums to 1. It involves the sum over all sequences that can possibly be mutated from *any* sequence that exists in the previous round. Since we start with only copies of the single wild-type sequence, the initial prevalence vector of sequences is 1 for the wild-type sequence w and 0 for all other sequences. If we know the fitness values $\pi(x)$ and the mutation probabilities $g(u \to x)$, then Equation (1) completely determines the prevalence of every sequence $x \in \Omega$ in every round r. We call the dynamics represented by Equation (1) the **infinite population dynamics**.

The dynamics of Equation (1) are hard to work with even when the fitness levels π are known. The equation (1) is not linear in the density $p^{(r)}(u)$, and the normalizing constant in the denominator involves a computationally intractable sum due to having to consider all sequences at the rth round as well as any possible sequence that can be mutated from sequences at the rth round. Facing such challenges, we propose to use approximate the infinite population dynamics in (1) via a linear dynamics which assumes competitions of fitness between sequences are localized among those which are mutated from the same direct ancestor. To illustrate, we consider an idealized experiment where for each sequence x at round r, an isolated environment is provided where only sequences which are mutated from x grow and compete in each environment during the $r+1$ round. At the end, all sequences are pooled together and the prevalence vector at the $r+1$th round $p^{(r+1)}$ is computed.

In this idealized experiment, the prevalence vector $p^{(r+1)}$ are given by

$$p^{(r+1)}(x) = \sum_{u\in\Omega} \frac{p^{(r)}(u) g(u \to x)\pi(x)}{\sum_{z\in\Omega} g(u \to z)\pi(z)}. \tag{2}$$

We can write this equation compactly in matrix form $p^{(r+1)} = S\, p^{(r)}$ where $S_{yx} := p(x \to y)$ where we recall that $p(x \to y)$ is defined as

$$p(x \to y) := \frac{g(x \to y)\pi(y)}{\sum_{z\in\Omega} g(x \to z)\pi(z)}. \tag{3}$$

We note $p^{(r+1)}$ is a valid probability vector because it is the product of a left stochastic matrix ($\sum_y S_{yx} = 1, \forall x$) and a stochastic column vector $p^{(r)}$. We call the dynamics represented by (3), **simplified Markov chain dynamics**, as we can think of it as arising from a Markov chain on the sequences of Ω. The transition probability between sequence x in round r and sequence y in round $r+1$ is given by $p(x \to y) := S_{yx}$.

The simplification from the infinite population dynamics (1) to the simplified Markov chain dynamics (2) is due to reducing many to many competitions to one to many competitions in the selection step (Figure S4), and therefore, the accuracy of approximation depends on abundance of sequences at each round as well as their relative fitness values. The approximation is expected to work well, for example, when there are relatively small number of distinct sequences as well as they share similar fitness properties, which is typically the case in neutral evolution experiment because introduced mutations tend to be neutral and a local neighborhood of the wild-type sequence is explored (Main, Fig. 1c). In the rest of the framework, we use the simplified Markov chain dynamics of (3) as an approximate model for the dynamics of the laboratory evolution experiment.

Approximate recursive relationships for first-order marginals

Let $S \subseteq [L]$ be a set of nodes which is of interest for the approximation of $\mu_S^{(r)}$. We approximate π with a distribution where the dependence between X_S and the remaining variables X_{S^c} is removed. In other words, we assume for any function f,

$$\mathbb{E}_\pi[f(X_{S^c})|X_S] \approx \mathbb{E}_\pi[f(X_{S^c})]. \tag{4}$$

Obviously, if π factorizes, the approximation (4) is exact for any $S \subseteq [L]$. In general, the accuracy of approximation depends on the strength of interactions between X_S and X_{S^c}.

$$\begin{aligned}
\mu_i^{(r+1)}(c) &= \sum_{x\in\Omega} p^{(r+1)}(x)\delta(x_i,c) \\
&= \sum_{x\in\Omega}\left\{\sum_{y\in\Omega} p^{(r)}(y)p(y\to x)\right\}\delta(x_i,c)(\text{by definition of } p^{(r+1)}(x)) \\
&= \sum_{x\in\Omega}\sum_{y\in\Omega} p^{(r)}(y)\cdot\frac{g(y\to x)\cdot\pi(x)}{\sum_{u\in\Omega} g(y\to u)\cdot\pi(u)}\cdot\delta(x_i,c)\,(\text{by definition of } p(x\to y)) \\
&= \sum_{y\in\Omega} p^{(r)}(y)\cdot\frac{\sum_{x\in\Omega} g(y\to x)\cdot\pi(x)\cdot\delta(x_i,c)}{\sum_{u\in\Omega} g(y\to u)\cdot\pi(u)}\ (\text{exchange of summation order}) \\
&= \sum_{y\in\Omega} p^{(r)}(y)\cdot\frac{g_i(y_i\to c)\,\mathbb{E}_\pi[\prod_{k\neq i} g_k(y_k\to X_k)\delta(X_i,c)]}{\sum_{c''\in\mathscr{C}} g_i(y_i\to c'')\,\mathbb{E}_\pi[\prod_{k\neq i} g_k(y_k\to U_k)\delta(U_i,c'')]}\ \text{where } X,U\sim\pi
\end{aligned} \tag{5}$$

Using the law of iterated expectations and $\mathbb{E}_\pi[\prod_{k\neq i} g_k(y_k\to X_k)|X_i] \approx \mathbb{E}_\pi[\prod_{k\neq i} g_k(y_k\to X_k)]$ by the assumption (4), we have,

$$\begin{aligned}
\mathbb{E}_\pi[\prod_{k\neq i} g_k(y_k\to X_k)\delta(X_i,c)] &= \mathbb{E}_\pi[\mathbb{E}_\pi[\prod_{k\neq i} g_k(y_k\to X_k)|X_i]\delta(X_i,c)] \\
&\approx \mathbb{E}_\pi[\prod_{k\neq i} g_k(y_k\to X_k)]\,\mathbb{E}_\pi[\delta(X_i,c)].
\end{aligned}$$

Therefore,

$$\begin{aligned}
\mu_i^{(r+1)}(c) &\approx \sum_{y\in\Omega} p^{(r)}(y)\cdot\frac{g_i(y_i\to c)\,\mathbb{E}_\pi[\prod_{k\neq i} g_k(y_k\to X_k)]\,\mathbb{E}_\pi[\delta(X_i,c)]}{\sum_{c''\in\mathscr{C}} g_i(y_i\to c'')\,\mathbb{E}_\pi[\prod_{k\neq i} g_k(y_k\to U_k)]\,\mathbb{E}_\pi[\delta(U_i,c'')]} \\
&= \sum_{y\in\Omega} p^{(r)}(y)\cdot\frac{g_i(y_i\to c)\mu_i(c)}{\sum_{c''\in\mathscr{C}} g_i(y_i\to c'')\mu_i(c'')}\ \text{since } \mu_i(c) = \mathbb{E}_\pi[\delta(V_i,c)] \text{ for } V\sim\pi \\
&= \sum_{c'\in\mathscr{C}}\frac{g_i(c'\to c)\mu_i(c)}{\sum_{c''} g_i(c'\to c'')\mu_i(c'')}\mu_i^{(r)}(c')
\end{aligned}$$

Note when $r=0$, $\mu_i^{(0)}(w_i)=1$ and $\mu_i^{(0)}(c')=0$ for $c'\neq w_i$. Therefore,

$$\mu_i^{(1)}(c) = \frac{g_i(w_i\to c)\mu_i(c)}{\sum_{c'} g_i(w_i\to c')\mu_i(c')}.$$

The derivation for the recursive relationships for pairwise marginals $\mu_{ij}^{(r)}$ are similar to the derivation above where we carry out similar calculations with $S=\{i,j\}$ instead of $S=\{i\}$, and is written in the next section.

Approximate recursive relationships for the second-order marginals

We have,

$$
\begin{aligned}
&\mu_{ij}^{(r+1)}(c,d) \\
&= \sum_{x\in\Omega} p^{(r+1)}(x)\delta\left(x_i,c\right)\delta\left(x_j,d\right) \\
&= \sum_{x\in\Omega}\left\{\sum_{y\in\Omega} p^{(r)}(y)\cdot p(y\to x)\right\}\delta\left(x_i,c\right)\delta\left(x_j,d\right)\text{(by definition of } p^{(r+1)}(x)) \\
&= \sum_{x\in\Omega}\sum_{y\in\Omega} p^{(r)}(y)\cdot\frac{g(y\to x)\cdot\pi(x)}{\sum_{u\in\Omega} g(y\to u)\cdot\pi(u)}\cdot\delta\left(x_i,c\right)\delta\left(x_j,d\right)\text{(by definition of } p(x\to y)) \\
&= \sum_{y\in\Omega} p^{(r)}(y)\cdot\frac{\sum_{x\in\Omega} g(y\to x)\cdot\pi(x)\cdot\delta\left(x_i,c\right)\delta\left(x_j,d\right)}{\sum_{u\in\Omega} g(y\to u)\cdot\pi(u)}\text{ (exchange of summation order)} \\
&= \sum_{y\in\Omega} p^{(r)}(y)\cdot\frac{\sum_{x\in\Omega} g(y\to x)\cdot\pi(x)\cdot\delta\left(x_i,c\right)\delta\left(x_j,d\right)}{\sum_{u\in\Omega}\sum_{c'',d''} g(y\to u)\cdot\pi(u)\cdot\delta\left(u_i,c''\right)\delta\left(u_j,d''\right)} \\
&= \sum_{y\in\Omega} p^{(r)}(y)\cdot\frac{\mathbb{E}_\pi\left[\delta\left(X_i,c\right)\delta(X_j,d)\,g(y\to X)\right]}{\sum_{c'',d''}\mathbb{E}_\pi\left[\delta\left(U_i,c''\right)\delta\left(U_j,d''\right)g(y\to U)\right]}\text{ where } X,U\sim\pi \\
&= \sum_{y\in\Omega} p^{(r)}(y)\cdot\frac{\mathbb{E}_\pi\left[\delta\left(X_i,c\right)\delta\left(X_j,d\right)g_i\left(y_i\to c\right)g_j\left(y_j\to d\right)\prod_{k\neq(i,j)} g_k(y_k\to X_k)\right]}{\sum_{c'',d''}\mathbb{E}_\pi\left[\delta\left(U_i,c''\right)\delta\left(U_j,d''\right)g_i\left(y_i\to c''\right)g_j\left(y_j\to d''\right)\prod_{k\neq(i,j)} g_k(y_k\to U_k)\right]} \qquad (6) \\
&\approx \sum_{y\in\Omega} p^{(r)}(y)\frac{g_i\left(y_i\to c\right)g_j\left(y_i\to d\right)\mathbb{E}_\pi\left[\delta\left(X_i,c\right)\delta\left(X_j,d\right)\right]\mathbb{E}_\pi[\prod_{k\neq(i,j)} g_k(y_k\to X_k)]}{\sum_{c'',d''} g_i\left(y_i\to c''\right)g_j\left(y_j\to d''\right)\mathbb{E}_\pi\left[\delta\left(U_i,c''\right)\delta\left(U_j,d''\right)\right]\mathbb{E}_\pi[\prod_{k\neq(i,j)} g_k(y_k\to U_k)]}. \qquad (7)
\end{aligned}
$$

where the equality in (6) is due to the definition $g(y\to x)=\prod_j g_j(y_j\to x_j)$, and the approximation in (7) is due to condition (4). Continuing to work with (7),

$$
\begin{aligned}
(7) &= \sum_{y\in\Omega} p^{(r)}(y)\frac{g_i\left(y_i\to c\right)g_j\left(y_i\to d\right)\mathbb{E}_\pi\left[\delta\left(X_i,c\right)\delta\left(X_j,d\right)\right]\mathbb{E}_\pi[\prod_{k\neq(i,j)} g_k(y_k\to X_k)]}{\sum_{c'',d''} g_i\left(y_i\to c''\right)g_j\left(y_j\to d''\right)\mathbb{E}_\pi\left[\delta\left(U_i,c''\right)\delta\left(U_j,d''\right)\right]\mathbb{E}_\pi[\prod_{k\neq(i,j)} g_k(y_k\to U_k)]} \\
&= \sum_{y\in\Omega} p^{(r)}(y)\frac{g_i\left(y_i\to c\right)g_j\left(y_i\to d\right)\mu_{ij}(c,d)}{\sum_{c'',d''} g_i\left(y_i\to c''\right)g_j\left(y_j\to d''\right)\mu_{ij}\left(c'',d''\right)} \qquad (8) \\
&= \sum_{y\in\Omega}\sum_{c'}\sum_{d'} p^{(r)}(y)\delta\left(y_i,c'\right)\delta\left(y_j,d'\right)\frac{g_i\left(c'\to c\right)g_j\left(d'\to d\right)\mu_{ij}(c,d)}{\sum_{c'',d''} g_i\left(c'\to c''\right)g_j\left(d'\to d''\right)\mu_{ij}\left(c'',d''\right)} \\
&= \mathbb{E}^{(r)}\left[\sum_{c'}\sum_{d'}\delta\left(Y_i,c'\right)\delta\left(Y_j,d'\right)\frac{g_i\left(c'\to c\right)g_j\left(d'\to d\right)\mu_{ij}(c,d)}{\sum_{c'',d''} g_i\left(c'\to c''\right)g_j\left(d'\to d''\right)\mu_{ij}\left(c'',d''\right)}\right]\text{ (where } Y\sim P^{(r)}) \\
&= \sum_{c'}\sum_{d'}\mu_{ij}^{(r)}(c',d')\frac{g_i\left(c'\to c\right)g_j\left(d'\to d\right)\mu_{ij}(c,d)}{\sum_{c'',d''} g_i\left(c'\to c''\right)g_j\left(d'\to d''\right)\mu_{ij}\left(c'',d''\right)}
\end{aligned}
$$

and the equality in (8) is due to $X\overset{d}{=}U$.

Estimating canonical parameters

Now, we describe how we convert the estimates for the mean parameters μ to the corresponding canonical parameters θ. In other words, we would like to obtain $\widehat{\theta}$ in the set of parameters Θ such that $E_{\widehat{\theta}}[\phi]=\widehat{\mu}$.

First of all, it is a well known fact that such $\widehat{\theta}$ exists in Θ if $\widehat{\mu}$ is in the interior of the set of valid mean parameters $\mathscr{M}$, although in general this mapping is not available in closed forms. The important exception is Gaussian Graphical Model (GGM) [6, 7] where for a normal distributed $z\in\mathbb{R}^p$ with mean m and variance Σ, i.e., $z\sim P_\theta$ such that

$\theta = (\{h_i\}_{i\in[p]}, \{e_{ij}\}_{i,j\in[p],i\le j})$ with $\mathbb{E}_\theta[z] = m, \mathrm{V}ar_\theta[z] = \Sigma$, we can explicitly derive the following mean parameters (m, Σ) to canonical parameters $\theta = (\eta, J)$ relationships:

$$\begin{aligned} f_\theta(z) &\propto \exp\{-\frac{1}{2}(z-m)^\top \Sigma^\dagger (z-m)\} \\ &\propto \exp\{z^\top \eta - \frac{1}{2} z^\top J z\}. \end{aligned}$$

where $J = \Sigma^\dagger \in \mathbb{R}^{p\times p}$ is a pseudo-inverse matrix of Σ and $\eta = \Sigma^\dagger m \in \mathbb{R}^p$. The use of the pseudo-inverse matrix is to handle the case where Σ is not of full-rank. We have $\Sigma^{-1} = \Sigma^\dagger$ if rank(Σ) = p.

In particular, when $z \in \{0,1\}^{Lq_a}$ is a one-hot encoding of a sequence, i.e., $z = [\{z_{(i,a)}\}_{i\in[L],a\in\mathscr{A}}]$ for $z_{(i,a)} = \delta(x_i, a)$, we have $z^2 = z$, and therefore,

$$\begin{aligned} f_\theta(z) &\propto \exp\{z^\top \eta - \frac{1}{2} z^\top J z\} \\ &= \exp\{\sum_{s\in[Lq_a]} (z_s \eta_s - \frac{1}{2} J_{ss} z_s^2) - \sum_{s,t\in[Lq_a],s<t} J_{st} z_s z_t\} \\ &= \exp\left\{\sum_{i\in[L]}\sum_{a\in\mathscr{A}} z_{(i,a)} h_i(a) + \sum_{i\in[L]}\sum_{i<j}\sum_{a,b\in\mathscr{A}} z_{(i,a)} z_{(j,b)} e_{ij}(a,b)\right\} \end{aligned}$$

where we let $h_s := (\eta_s - \frac{1}{2}J_{ss})$, $e_{st} := -J_{st}$ for $s,t \in [Lq_a], s<t$, and $h_i(a)$ and $e_{ij}(a,b)$ refers to (i,a)th and $(i,a),(j,b)$th element of h and e.

In the following, we will utilize the relationships $h_s = (\Sigma^\dagger m)_s - \frac{1}{2}\Sigma^\dagger_{ss}$ and $e_{st} = -\Sigma^\dagger_{st}$ to estimate corresponding canonical parameters from the estimated mean parameters. First of all, we compute the estimated covariance matrix for $\Sigma := \mathrm{Var}_\theta(z) \in \mathbb{R}^{Lq_a\times Lq_a}$ between the first order sufficient statistics based on the estimated $\widehat{\nu}$. We choose the states that are the least frequent for each position as reference states. For each $i,j \in [L]$ and $a,b \in \mathscr{A}$, we have,

$$\widehat{\Sigma}((i,a),(j,b)) = \begin{cases} \widehat{\nu}_{ij}(a,b) - \widehat{\nu}_i(a)\widehat{\nu}_j(b) & \text{if } i \neq j \\ \widehat{\nu}_i(a) - \widehat{\nu}_i(a)^2 & \text{if } i = j, a = b \\ -\widehat{\nu}_i(a)\widehat{\nu}_i(b) & \text{if } i = j, a \neq b \end{cases}$$

If the estimated mean vector $\widehat{\nu}$ is globally consistent, then the resulting $\widehat{\Sigma}$ would be semi-positive definite. However, since we solved a relaxed problem in Main equation (9), the estimated ν is not necessarily globally consistent. One consequence of this is that $\widehat{\Sigma}$ may have negative eigenvalues. We carry out a regularized inference to estimate (h,e). Specifically, we let

$$\widehat{\Sigma}_\lambda = (\widehat{\Sigma} + \lambda \mathrm{I}), \tag{9}$$

and obtain $\{\widehat{h}_i(a)\}_{i\in[L],a\in\mathscr{A}}$ and $\{\widehat{e}_{ij}(a,b)\}_{i,j\in[L],i<j,a,b\in\mathscr{A}}$ using

$$\widehat{h} = \widehat{\Sigma}_\lambda^{-1}\widehat{\nu}_1 - \frac{1}{2}\mathrm{diag}(\widehat{\Sigma}_\lambda^{-1}) \quad \text{and} \quad \widehat{e} = -\widehat{\Sigma}_\lambda^{-1}, \tag{10}$$

where we define $\widehat{\nu}_1 := [\{\widehat{\nu}_i(a)\}_{i\in[L],a\in\mathscr{A}}]$ as the collection of first-order mean vectors at the amino-acid levels.

Finally, we estimate the Potts model energy function $\mathscr{E}(x)$ for a sequence $x = [x_1,\ldots,x_L]$ as follows

$$\widehat{\mathscr{E}}_\lambda(x) := -\left(\sum_{i\in[L]} \widehat{h}_i(AC(x_i)) + \sum_{i\in[L]}\sum_{i<j} \widehat{e}_{ij}(AC(x_i), AC(x_j))\right).$$

We use this energy function to design new proteins.We note the estimation procedure via regularizing diagonals (9),(10) is valid in the following sense: if $\|\widehat{\Sigma} - \Sigma\|_2 \to 0$, $\widehat{\mathscr{E}}_\lambda(x) \to \mathscr{E}(x)$ and $\widehat{e}_{ij}(a,b) \to e_{ij}(a,b)$ as $\lambda \to 0$, for any x and (i,j) such that $i<j$.

Additional Framework assumptions

In addition to the important assumptions mentioned in Main (Sec. Discussion), we also assume that the growth time for the population each round should be consistent from round to round. This is so that the fitness function $\pi(x)$ represents number of number of copies of a sequence for a consistent amount of time across rounds. This growth time for a bacterial population could be anywhere in the Exponential, Stationary or Death phases. Depending on the growth time selected, the inferred fitness landscape could exhibit different levels of epistasis.

References

1. Hopf, T. A. *et al.* Mutation effects predicted from sequence co-variation. *Nature biotechnology* **35,** 128–135 (2017).
2. Frazer, J. *et al.* Disease variant prediction with deep generative models of evolutionary data. *Nature* **599,** 91–95 (2021).
3. Stiffler, M. A. *et al.* Protein structure from experimental evolution. *Cell Systems* **10,** 15–24 (2020).
4. Eigen, M. & Schuster, P. A principle of natural self-organization. *Naturwissenschaften* **64,** 541–565 (1977).
5. Wilke, C. O. Quasispecies theory in the context of population genetics. *BMC evolutionary biology* **5,** 1–8 (2005).
6. Jones, D. T., Buchan, D. W., Cozzetto, D. & Pontil, M. PSICOV: precise structural contact prediction using sparse inverse covariance estimation on large multiple sequence alignments. *Bioinformatics* **28,** 184–190 (2012).
7. Dauparas, J. *et al.* Unified framework for modeling multivariate distributions in biological sequences. *arXiv preprint arXiv:1906.02598* (2019).
