# Supplementary material for: Inferring protein fitness landscapes from laboratory evolution experiments
Source: PLoS Comput Biol. 2023 Mar 1;19(3):e1010956. doi: 10.1371/journal.pcbi.1010956 (PMC10010530; doi:10.1371/journal.pcbi.1010956)
Supplement: S2 Text — (PDF) [file pcbi.1010956.s011.pdf]

VAE architecture and hyperparameters

The latent space of a two dimensional variational auto encoder (VAE) is used to visualize natural DHFR sequences in Main Fig 1(c). The VAE decoder has a fully connected hidden linear layer with dimension 400 and sigmoid activation functions. The latent layer (bottle neck) is dimension 2. The VAE encoder mirrors the VAE decoder with a hidden Linear layer of dimension 400. The learning rate used is 0.001 and the model is trained for 300 epochs with a batch size of 128. Custom scripts were implemented in Python using PyTorch.
